# Capsaicin modulates Akkermansia muciniphila abundance by enhancing MUCIN2 levels in mice fed with high-fat diets

**DOI:** 10.29219/fnr.v67.9990

**Published:** 2024-04-17

**Authors:** Ting Gong, Yujing Zhou, Qinhong Shi, Yanyan Li, Haizhu Wang, Min Zhang, Linzheng Liao

**Affiliations:** 1 Chongqing college of tradtional chinese medicine, school of general eduction, Chongqing, China; 2 Chongqing Medical and Pharmaceutical College, College of nursing, Chongqing, China; 3 Chongqing University of Arts and Science, Horticultural Plant Engineering Research Center of Chongqing Universities, Chongqing, China

**Keywords:** *capsaicin*, *TRPV1*, Akkermansia muciniphila, *MUC2*

## Abstract

Extensive research has been conducted to investigate the impact of capsaicin (CAP) on lipid metabolism, focusing specifically on its interaction with the vanilloid subtype 1 (TRPV1) ion channel. Additionally, studies have illuminated the role of Akkermansia muciniphila (*A. muciniphila*), a specific strain of intestinal microbiota, in lipid metabolism. In this study, a model utilizing resiniferatoxin (RTX) was employed to deactivate TRPV1 ion channels in germ-free mice, followed by the administration of A. muciniphila via gavage. Following the collection of intestinal tissues for a comprehensive analysis, employing histopathology, qPCR, and ELISA techniques, our findings revealed a significant upregulation of MUC2 and MUC3 expression induced by CAP. This upregulation resulted in the thickening of the colonic mucus layers. Notably, this effect was absent when TRPV1 was selectively inhibited. Moreover, there was no discernible impact on goblet cells. The findings strongly indicate that CAP influences the system by activating the TRPV1 ion channel, thereby enhancing the expression of mucin MUC2 and promoting an augmentation in the thickness of the mucous layer. This activation, in turn, supplies *A. muciniphila* with an ample source of carbon and nitrogen. This insight potentially clarify the underlying mechanism through which CAP facilitates the increase in *A. muciniphila* abundance.

## Popular scientific summary

- Capsaicin enhances the secretion of colonic mucin MUC2 through TRPV1, providing ample carbon and sources for A. muciniphila, promotes its proliferation, and thus regulates abnormal lipid metabolism.

Numerous scientific investigations have extensively explored the robust correlation between intestinal microorganisms and metabolic irregularities triggered by a high-fat diet. Intestinal microorganisms have recently emerged as a novel focal point for modulating abnormal lipid metabolism (1). Specifically, *Akkermansia muciniphila* (*A. muciniphila*), a Gram-negative anaerobic bacterium categorized in the *Akkermansia* genus of the *Verrucomicrobia* phylum, has gained recognition (2). This bacterium is widely acknowledged for its role in mucin degradation and is heralded as a next-generation probiotic capable of regulating glucose metabolism, mitigating low-grade inflammation, restraining fat accumulation, and ameliorating lipid metabolism disorders (1, 3–5).

Capsaicin (CAP), a naturally occurring compound found in select foods, has been implicated in the regulation of abnormal lipid metabolism. Numerous studies have delved into the mechanisms through which CAP influences abnormal lipid metabolism. The transient receptor potential vanilloid subtype 1 (TRPV1) ion channel, acting as a sensor for CAP, has been reported to participate in the regulation of lipid metabolism (6). However, recent research has revealed that CAP-induced alterations in intestinal microorganisms result in increased abundance of *A. muciniphila* and other bacterial strains, along with elevated levels of short-chain fatty acids (7–9). Nevertheless, the precise mechanism underlying CAP’s promotion of increased *A. muciniphila* abundance remains elusive.

*A. muciniphila* is an anaerobic chemo-organotroph that relies exclusively on mucin as its source of nitrogen, carbon, and energy (10–12). Consequently, our study sought to elucidate the mechanisms driving the heightened abundance of *A. muciniphila* induced by CAP in high-fat diet-induced obese mice through an evaluation of mucin levels.

## Materials and methods

### Materials

CAP (≥95%) was acquired from Henan bis-biotech Co., Ltd., China. *A. muciniphila* ATTC BAA-835 was procured from the American Type Culture Collection.

### Animals and treatments

A total of 50 male specific germ-free Kunming mice, aged 6–8 weeks and weighing 25–40 g, were obtained from the Laboratory Animal Center of Army Medical University in China. The Institutional Animal Care and Use Committee of Army Medical University (AMUWEC20226157) approved the experimental protocols. Germ-free KM Tac mice were bred and housed in plastic gnotobiotic isolators at Army Medical University. The mice were individually housed in cages maintained at a temperature of 25 ± 1°C, with a relative humidity of 40–70% and subjected to a 12-h light/12-h dark cycle. The experiment spanned 4 weeks. Prior to the commencement of the experiment, the mice were provided unrestricted access to water and a standard diet for 1 week. Subsequently, the mice were randomly divided into four groups (*n* = 10): the high-fat diet group (HF), the high-fat diet group supplemented with 0.01% CAP (HC), the high-fat diet + 0.01% CAP + resiniferatoxin (RTX) (200 μg/kg body weight of the animal) (HCR), the high-fat diet + 0.01% CAP +2×10^8^ CFU A. muciniphila (HCA), and the high-fat diet + 0.01% CAP+RTX (200 μg/kg body weight of the animal) + 2×10^8^ CFU A. muciniphila (HCRA). CAP was integrated into the mice’s chow, with the dosage selection based on rele-vant studies (8, 13). The composition of the diets for the experimental groups is detailed in Supplementary Table S1. The high-cholest erol feed included a 1% cholesterol supplement in accor dance with the US Standard AIN-93 feed formula (14).

### Sample collection

Throughout the trial, daily monitoring was conducted to assess animal mortality. The mice’s body weight and food consumption were documented on a weekly basis. At the end of the 4-week period, the mice were euthanized to facilitate the collection of blood samples, along with samples from the colon, cecum, small intestine, and their respective contents. Blood samples were collected through eyeball extraction. The remaining samples were promptly placed in germ-free containers and flash-frozen in liquid nitrogen for 30 sec. Subsequently, they were stored at -80°C until further analysis. All procedures were executed meticulously under aseptic conditions.

### Chemodenervation and confirmation tests

Successful ablation of TRPV1+ neurons in the HR and HCR group animals was achieved through a single intraperitoneal injection of the maximum tolerable dose of RTX (200 μg/kg body weight). The confirmation of effective ablation was based on the absence of observed physiological responses after 24 h, following a method adapted from prior literature with minor adjustments (15). To validate the ablation, two tests were conducted: the hot plate test and the eye wipe test. In the hot plate test, the surface temperature was meticulously maintained at 60 ± 5°C, and the number of jumps or paw licks within a 15-sec interval was documented. Each animal underwent the test three times, with a 5-min interval between repetitions. The eye wipe test entailed the administration of a 0.02% (w/v) CAP solution, and the number of eye wipes within a 30-sec period was recorded. Replicates were independently taken from each eye of the animal.

### Mucin2 analysis

The levels of MUC2 in the colon were measured using an enzyme-linked immunoassay (ELISA) kit (Sinobestio, Shanghai).

### Histopathologic analysis

After fixation in 10% buffered formalin, the colon tissue samples underwent a series of steps. They were dehydrated using various ethanol concentrations, followed by clearing in xylene. Subsequently, the samples were embedded in paraffin blocks and sliced into approximately 5 µm thick sections. These sections were then subjected to staining with Periodic Acid-Schiff (PAS) to facilitate histopathological assessment and visualization of the thickness of the colonic mucus layer. To visualize the goblet cells in the colon tissue, a combination of Alcian Blue (AB)-PAS stain and nuclear fast red (Sigma-Aldrich) staining techniques was employed. Microphotographs of all the histological slides were captured using a light imaging microscope (BX40F4, NIKON, Tokyo, Japan). Finally, the mean thickness was calculated using an image analysis program.

### RNA isolation and quantitative real-time polymerase chain reaction

Total RNA was extracted from the colon tissue of the mice using the Ribopure RNA extraction kit (Invitrogen, USA), following the manufacturer’s instructions. For cDNA synthesis, 1 μg of total RNA was employed with a RT-PCR kit (Invitrogen, Rockville, MD) and the SuperScript III First-Strand Synthesis System. The resulting cDNA derived from the total RNA was analyzed using a real-time quantitative PCR system (CFX96 Touch™ Real-Time PCR Detection System, Bio-Rad, USA). The specific primers used for the genes of interest in this study are listed in Supplementary Table S2. The relative gene expression values were determined using the ∆∆Ct method (∆∆Ct = ∆∆Ct treated − ∆∆Ct untreated control), and the equation y = 2^−∆∆Ct was applied. β-Actin served as the reference gene, and mRNA levels in different experimental groups were compared to those of the control group, which were normalized to 1 (16).

### Statistical analysis

The data were presented as mean ± standard deviation (*n* = 8). Statistical analysis was conducted using Origin 8.5 and SPSS version 20.0. One-way analysis of variance (ANOVA) was utilized, followed by Duncan’s multiple-range test to evaluate differences among the groups. A significance level of *P* < 0.05 was considered statistically significant.

## Results

### Behavioral validation of selective ablation of TRPV1

**Fig. 1:**
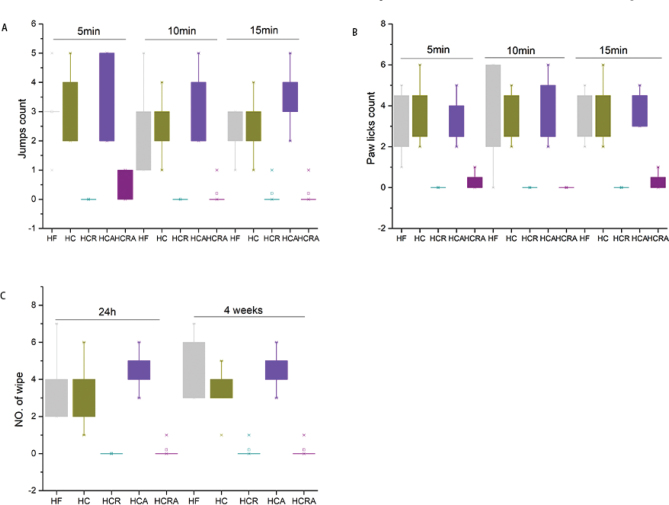
(A–C) Behavioral pain assays for validating TRPV1 denervation in RTX-treated mice. (A–B) Hot plate test; (C) Eye wipe test.

In mice, the administration of RTX led to the complete elimination of physiological responses to noxious heat and CAP. During the hot plate test, neither jumping nor paw licking behaviors were observed in the HCR and HCRA groups (Fig. 1A and B), indicating a complete insensitivity to temperatures (60 ± 5°C). These findings validate the systemic ablation of TRPV1 receptors in the treated animals. To evaluate the response to CAP-induced pain, a 0.02% (w/v) CAP solution was employed in the eye wipe test. Similarly, no response was observed in the HCR and HCRA groups, while the control group exhibited frequent eye wiping (Fig. 1C). These results are consistent with previous studies on TRPV1 denervation using RTX. Furthermore, these consistent outcomes persisted in the eye wipe test even after a 4-week period (Fig. 1C), confirming the absence of significant recovery of TRPV1+ neurons.

### Effect of CAP on weight gain and food intake of germ-free mice

**Fig. 2:**
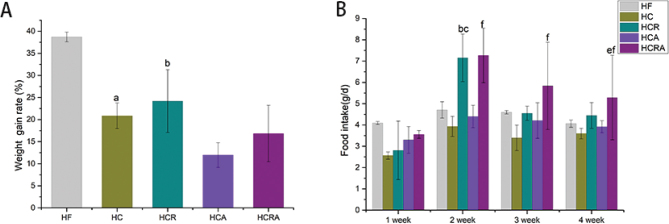
Effects of CAP on body weight (A) and feed intake (B) of germ-free mice in different treatment groups. Note: Values are expressed as mean ± standard deviation (*n* = 10). ‘a’ denotes a significant difference between the HF and HC groups. ‘b’ denotes a significant difference between the HF and HCR groups. ‘c’ denotes a significant difference between the HC and HCR groups. ‘d’ denotes a significant difference between the HC and HCA groups. ‘e’ denotes a significant difference between the HCR and HCRA groups. ‘f’ denotes a significant difference between the HCA and HCRA groups (Dunnett test after one-way ANOVA, *P* < 0.05).

The body weight of the HC and HCR groups decreased by 53.7 and 48.9%, respectively, in comparison to the HF group (Fig. 2A). Two weeks later, the HC group displayed notably reduced food intake in comparison to the HCR group. Moreover, the HCRA group exhibited increased food intake compared to the HCA group.

### Effect of CAP on the number of colonic goblet cells of germ-free mice in different treatment groups

**Fig. 3:**
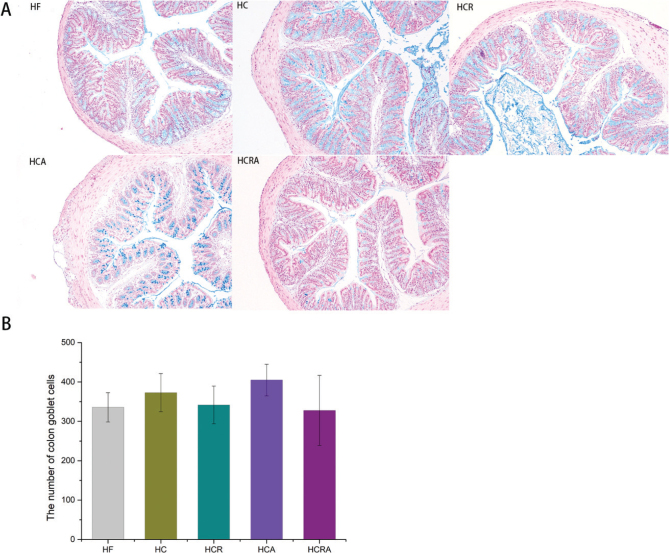
(A) AB-PAS staining (100×) of goblet cells in colon tissue. (B) Quantification of goblet cells in colonic tissue. Note: Values are expressed as mean ± standard deviation (*n* = 10) (Dunnett test after one-way ANOVA, *P* < 0.05).

Figure 3 presents the histopathological analysis of excised tissue from various experimental groups. In the HF, HC, and HCR groups, the intestinal structure appeared normal, with no reduction in the number of crypts in the mucosal layer. The epithelial cells displayed a well-organized and tightly packed arrangement, and goblet cells were abundant. Similarly, in the HCA group, the intestinal structure was largely normal, with no decrease in the number of crypts in the mucosal layer. The epithelial cells exhibited a well-arranged and tightly packed pattern, without any noticeable degeneration or shedding. Furthermore, there was a significant presence of goblet cells within the crypts. In the HCRA group, the intestinal structure also appeared normal, with no reduction in the number of crypts in the mucosal layer. The epithelial cells were neatly arranged and tightly packed, showing no signs of denaturation, and a substantial number of goblet cells were observed (Fig. 3A). The quantification of goblet cells in the visual field was performed using Image-Pro Plus 6.0 software. Statistical analysis revealed that the number of goblet cells in the HCA group did not exhibit significant changes, but it was slightly higher compared to the other groups (Fig. 3B).

### Effect of CAP on mucous layer of germ-free mice in different treatment groups

**Fig. 4:**
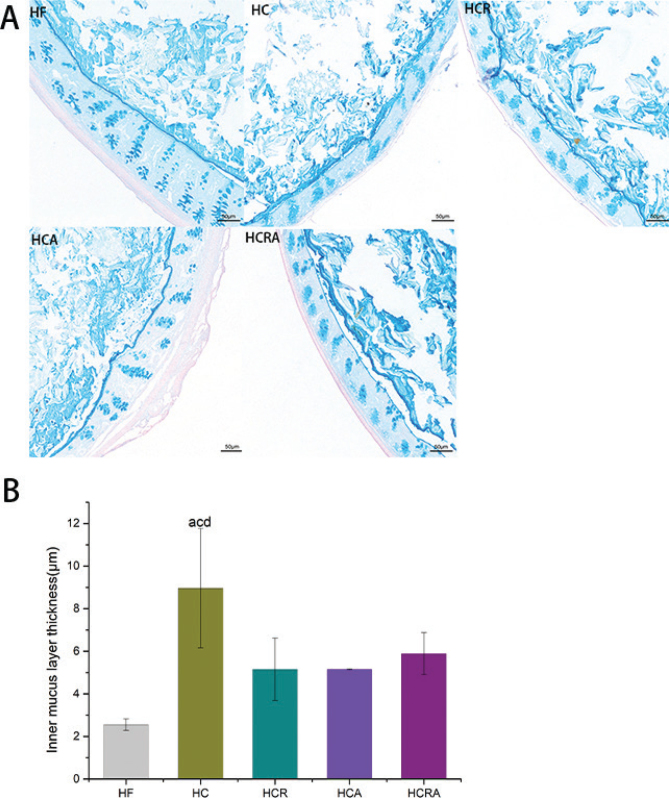
(A) PAS staining of the mucous layer. (B) Analysis of the thickness of the mucous layer. Note: Values are expressed as mean ± standard deviation (*n* = 10). ‘a’ denotes a significant difference between the HF and HC groups. ‘b’ denotes a significant difference between the HF and HCR groups. ‘c’ denotes a significant difference between the HC and HCR groups. ‘d’ denotes a significant difference between the HC and HCA groups. ‘e’ denotes a significant difference between the HCR and HCRA groups (Dunnett test after one-way ANOVA, *P* < 0.05).

Figure 4A illustrates the visualization of mucous layer thickness in the inner colon wall using PAS staining. In Fig. 4B, it is evident that the inclusion of CAP led to a notable increase in mucous layer thickness in the HC group by 8.96 μm. In contrast, following the selective ablation of TRPV1 by RTX, the mucous layer thickness in the HCR group significantly decreased to 5.15 μm. The mucous layer thickness in the HCA, HCR, and HCRA groups was notably lower compared to the HC group, with no significant differences observed among the HCA, HCR, and HCRA groups.

### Effect of CAP on colonic mucin content of germ-free mice in different treatment groups

**Fig. 5:**
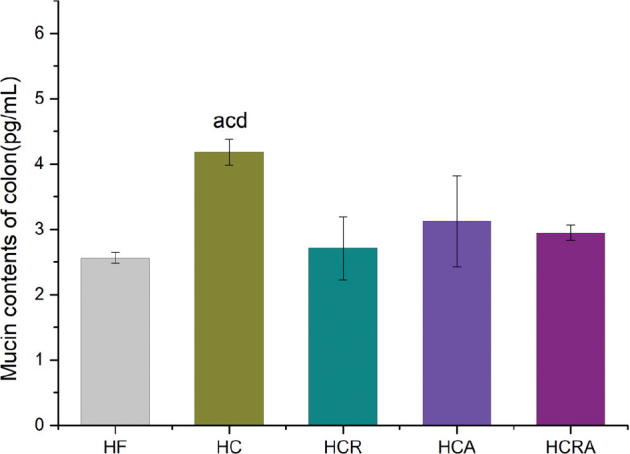
Effects of CAP on colonic mucin content of germ-free mice in different treatment groups. Note: Values are expressed as mean ± standard deviation (*n* = 10). ‘a’ denotes a significant difference between the HF and HC groups. ‘b’ denotes a significant difference between the HF and HCR groups. ‘c’ denotes a significant difference between the HC and HCR groups. ‘d’ denotes a significant difference between the HC and HCA groups. ‘e’ denotes a significant difference between the HCR and HCRA groups (Dunnett test after one-way ANOVA, *P* < 0.05).

According to the results depicted in Fig. 5, the mucin content in the HC group displayed a substantial increase of 63.3% when compared to the HF group, and a 51.3% increase in comparison to the HCR group. Furthermore, there was a significant elevation of 33.9% in mucin content in the HC group when contrasted with the HCA group. Although the HCRA group exhibited a marginal increase in mucin content in comparison to the HCR group, this difference did not achieve statistical significance.

### Effect of CAP on genes related to colonic mucin secretion of germ-free mice in different treatment groups

**Fig. 6:**
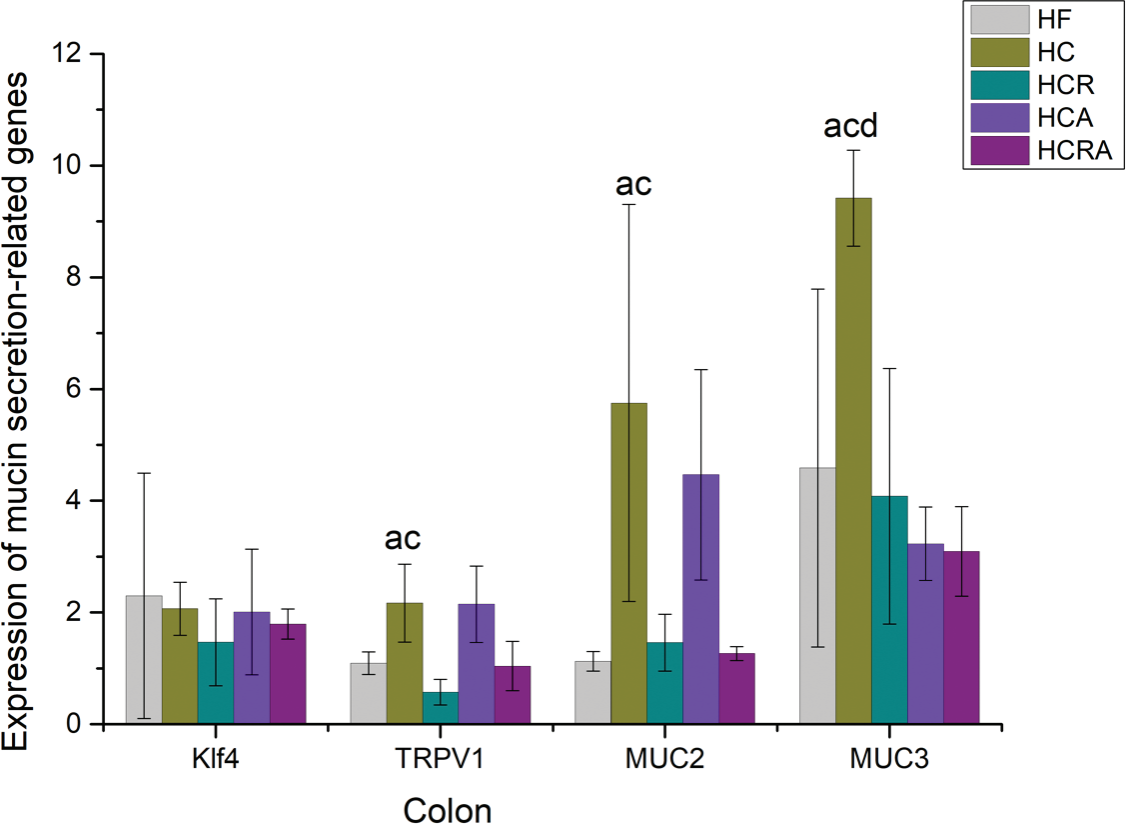
Effect of CAP on genes related to colonic mucin secretion of germ-free mice in different treatment groups. Note: Values are expressed as mean ± standard deviation (*n* = 10). ‘a’ denotes a significant difference between the HF and HC groups. ‘b’ denotes a significant difference between the HF and HCR groups. ‘c’ denotes a significant difference between the HC and HCR groups. ‘d’ denotes a significant difference between the HC and HCA groups. ‘e’ denotes a significant difference between the HCR and HCRA groups (Dunnett test after one-way ANOVA, *P* < 0.05).

Figure 6 illustrates the impact of CAP on genes associated with mucin secretion in the colons of germ-free mice across various treatment groups. The expression of Klf4 in the colon did not reveal any significant differences among the groups. However, TRPV1 expression in the HC group exhibited a substantial increase, with a 99.1% elevation compared to the HF group, a 280.7% increase compared to the HCR group, and a 107.7% increase compared to the HCRA group. Furthermore, in comparison to the HF group, Muc2 expression in the HC group displayed a significant increase of 339.1%, while the HCR group demonstrated a notable increase of 280.4%. Additionally, MUC3 expression in the HC group was significantly higher than that in the HF, HCR, and HCA groups.

## Discussion

*A. muciniphila* is a bacterium found in the human intestinal tract, first isolated from stool samples in 2004. Its distinctive capability to facilitate the degradation of mucin within the gut positions it as a crucial organism residing at the interface between the intestinal lumen and host cells. Despite its relatively recent discovery, *A. muciniphila* stands as the sole cultured representative of the Verrucomicrobia phylum, readily detectable in phylogenetic and metagenomic analyses, thereby distinguishing it as one of the prominent phyla in the human gut (17). Reduced levels of *A. muciniphila* have been consistently noted in individuals with inflammatory bowel disease, notably ulcerative colitis, and those with metabolic disorders, implying its potential anti-inflammatory properties (18). Furthermore, *A. muciniphila* has exhibited beneficial effects across various pathological conditions, encompassing lipid reduction, inflammation mitigation, delayed aging, enhanced immunity, and the regulation of the nervous system (19).

Multiple studies have documented the ability of CAP to elevate the abundance of *A. muciniphila* in mice (7, 8). It has been reported that CAP’s impact on *A. muciniphila* abundance is mediated by the TRPV1 channels (20). Consequently, we selectively incapacitated TRPV1 ion channels via intraperitoneal injection of RTX. Subsequently, we confirmed the successful modeling of the HCR and HCRA groups by observing their unresponsiveness to heat and pain stimuli during the hot plate experiment at 60 ± 5°C and the CAP-induced ocular discomfort experiment. In contrast, the relevant control group exhibited conspicuous jumping and eye rubbing behaviors in response to both stimuli. Hence, our modeling results align with the findings reported by Kumar et al. (15). In terms of body weight, we observed a notable decrease in the body weight of mice in both the HC and HCR groups. However, the feed intake of the HCR and HCRA groups exhibited a significant increase compared to that of the HC group. This heightened feed consumption may be attributed to the stimulated desensitization of CAP in mice following the selective ablation of TRPV1 by RTX.

The colon epithelium primarily consists of closely connected cells, including columnar, cup-shaped, and enteric endocrine cells, representing differentiated epithelial lineages (21). Goblet cells play a crucial role in mucin secretion, forming the physical barrier of the colonic mucosa (22). Therefore, we conducted a histopathological analysis to evaluate the number of colonic goblet cells in germ-free mice subjected to selective TRPV1 ablation. The analysis revealed no significant differences in the number of goblet cells among the treatment groups. This finding suggests that CAP and selective ablation of TRPV1 have no impact on the goblet cell count, consistent with the results reported by Shen Wei et al. (20).

The mucous layer functions as a critical defense mechanism, safeguarding the intestinal lining against microorganisms and other luminal compounds. In the colon, it assumes an additional role as it harbors a distinct microbial community known as the mucus-associated microbiome (23). This microbial community has evolved alongside the host over an extended period, establishing a mutually beneficial relationship (24). The mucus layer contributes to various host functions, including facilitating efficient mucin turnover and stimulating goblet cells through toll-receptor mediation to promote mucus production (25). Continuous mucus production is essential for maintaining the integrity of the intestinal barrier and can inhibit the growth of pathogenic bacteria by generating antimicrobial compounds and promoting the growth of beneficial bacteria (26). Moreover, the mucus-associated microbiota confers ‘colonization resistance’ by occupying the pathogenic niche with symbiotic species and producing metabolites that interact with both the microbes and the host, significantly influencing the development of the host immune system (27).

*A. muciniphila* produces several mucolytic enzymes that degrade mucus, serving as its primary carbon and nitrogen source (3). Consequently, it is considered a key member of the mucus-associated flora (28). In our analysis of the mucous layer in mice from different treatment groups, we observed a significant difference in the thickness of the mucous layer between the HC and HCR groups, suggesting that CAP may promote mucous layer secretion. Conversely, the mucous layer in the HCA group was significantly thinner than that in the HC group, possibly due to *A. muciniphila* utilizing the mucous layer as a nitrogen source. Although the difference between the HCRA and HCR groups did not reach statistical significance, there was a slight increase in mucous layer thickness in the HCRA group compared to the HCR group, which could be attributed to the inherent ability of *A. muciniphila* to stimulate mucous layer growth (29).

The intestinal mucus layer primarily consists of Mucin 2 (MUC2), a highly glycosylated gel produced by goblet cells (30). Alongside epithelial cells, MUC2 forms a protective barrier against harmful substances. It constitutes a major component of the colonic mucosal layer, known for its role in inhibiting intestinal inflammation and preventing the formation and progression of intestinal tumors (31–33). *A. muciniphila*, a probiotic bacterium, is recognized for its ability to degrade human intestinal mucin (15). It possesses more than 300 genes that potentially participate in mucin degradation (34). A recent study identified a secreted protein from *A. muciniphila*, named Amuc_1434, capable of breaking down MUC2 (3). Consequently, we assessed the levels of MUC2 in the colon. Our findings indicated that the MUC2 levels in the HC group were significantly higher than those in the HF group, suggesting that CAP may promote the increase of MUC2, corroborating the results reported by Shen Wei et al. (20). When CAP was administered to mice with selective TRPV1 ablation, we observed a notable decrease in MUC2 levels compared to the HC group, implying that the inactivation of TRPV1 ion channels might hinder MUC2 secretion. Nevertheless, both the HCA and HCRA groups exhibited a decrease in MUC2 levels compared to the HC group, likely attributable to the depletion of MUC2 induced by the colonization of *A. muciniphila.*

*KLF4*, a transcription factor containing zinc finger motifs, regulates the formation and differentiation of colonic goblet cells during mouse development (35). We found no significant difference in *KLF4* expression in the colon among the various treatment groups, indicating that CAP did not exert a significant influence on goblet cell differentiation. Our results demonstrated that CAP can stimulate the expression of *MUC2* and *MUC3* genes in the colon. Furthermore, the stimulatory effect of CAP on *MUC2* and *MUC3* expression substantially diminished after TRPV1 elimination using RTX, suggesting that TRPV1 may play a pivotal role in CAP-induced expression of *MUC2* and MUC3 in the colon. These findings align with our prior observations regarding mucin content. In summary, these results suggest that CAP enhances the secretion of colonic mucin MUC2 through TRPV1, providing ample carbon and nitrogen sources for *A. muciniphila* and thereby promoting its proliferation.

## Conclusions

The current study presents robust evidence that CAP can augment mucous layer thickness and elevate the expression of mucin MUC2 through TRPV1 ion channel activation. This activation, in turn, affords *A. muciniphila* an abundant supply of carbon and nitrogen, shedding light on the mechanism underlying CAP’s promotion of increased *A. muciniphila* abundance. Moreover, our findings reveal a slight decrease in body weight in germ-free mice subsequent to *A. muciniphila* administration, aligning with prior research suggesting an inverse relationship between *A. muciniphila* and weight gain (36).

## Declaration of competing interest

All authors declare that they have no conflict of interest.

## Subsection ethics statement

All procedures followed were in accordance with the ethical standards of the responsible committee on human experimentation Institutional Animal Care and Use Committee of Southwest University.

## Supplementary Material


