# Supplementary material for: Capsaicin modulates Akkermansia muciniphila abundance by enhancing MUCIN2 levels in mice fed with high-fat diets
Source: Food Nutr Res. 2024 Apr 17;67:10.29219/fnr.v67.9990. doi: 10.29219/fnr.v67.9990 (PMC11077401; doi:10.29219/fnr.v67.9990)
Supplement: Supplementary file 1 [file FNR-67-9990-s1.docx]

**Table S1** The ingredient compositions of the experimental diets (g/kg Diet)

| Ingredients | HF | HC |
| --- | --- | --- |
| Corn starch | 449.5 | 449.5 |
| Lard | 100 | 100 |
| Soybean oil | 40 | 40 |
| a AIN-76 mineral mixture (%mixture) | 35 | 35 |
| Casein | 200 | 200 |
| b AIN-76 vitamin mixture (%mixture) | 10 | 10 |
| Sucrose | 100 | 100 |
| L-cystine | 3 | 3 |
| Cellulose | 50 | 50 |
| Choline Chloride | 2.5 | 2.5 |
| Cholesterol | 10 | 10 |
| Capsaicin |  | 0.1 |

**TableS2** Sequences of primers used for RT-qPCR

| **Gene** | **Sequence（5’ to 3’）** |
| --- | --- |
| Mu-GAPDH-F | AGGTCGGTGTGAACGGATTTG |
| Mu-GAPDH-R | TGTAGACCATGTAGTTGAGGTCA |
| Mu-Klf4-F | GTAGTGCCTGGTCAGTTCATC |
| Mu-Klf4-R | AACCTATACCAAGAGTTCTCATCTC |
| Mu-TRPV1-F | GGTTGTGACTTTCCCCTTC |
| Mu-TRPV1-R | TGCATGTCCTTTGAGTGTG |
| Mu-MUC2-F | ACATGGATGGCTGCTTCT |
| Mu-MUC2-R | TGCTCACAGTCGTTGGTAA |
| Mu-MUC3-F | GGGAAAACTGCGACTGG |
| Mu-MUC3-R | GGATGGAGAACACGAGGA |
